# Investigating the Short-Term Effects of Cold Stress on Metabolite Responses and Metabolic Pathways in Inner-Mongolia Sanhe Cattle

**DOI:** 10.3390/ani11092493

**Published:** 2021-08-25

**Authors:** Lirong Hu, Luiz F. Brito, Zaheer Abbas, Abdul Sammad, Ling Kang, Dongsheng Wang, Hongjun Wu, Airong Liu, Guiqiang Qi, Man Zhao, Yachun Wang, Qing Xu

**Affiliations:** 1 College of Life Sciences and Bioengineering, Beijing Jiaotong University, Beijing 100044, China; 15121578@bjtu.edu.cn (L.H.); zaheerabbas@bjtu.edu.cn (Z.A.); 14121580@bjtu.edu.cn (L.K.); 2 Key Laboratory of Animal Genetics, Breeding and Reproduction, MARA, National Engineering Laboratory for Animal Breeding, Beijing Engineering Technology Research Center of Raw Milk Quality and Safety Control, College of Animal Science and Technology, China Agricultural University, Beijing 100193, China; drabdulsammad1742@yahoo.com; 3 Department of Animal Sciences, Purdue University, West Lafayette, IN 47907, USA; britol@purdue.edu; 4 Xiertala Cattle Breeding Farm, Hailaer Farm Buro, Hailaer, Hulunbuir 021012, China; 15334801769@163.com (D.W.); 15334801820@163.com (H.W.); xumulingcanbu@sina.com (A.L.); qgq15334801911@163.com (G.Q.); zm653596759@163.com (M.Z.)

**Keywords:** cold stress, Sanhe cattle, metabolomics, climatic resilience

## Abstract

**Simple Summary:**

Cold stress is a major environmental stressor affecting cattle performance in temperate regions, which causes impaired welfare and economic losses to cattle producers. The identification of biological mechanisms associated with cold stress response is paramount for developing effective mitigation strategies, such as genomic selection. In this study, we assessed the short-term effects of hyper-cold stress on metabolite responses and metabolic pathways in the serum of Inner-Mongolia Sanhe cattle. Moreover, 19 differential metabolites were found, mainly involved in amino acid metabolism. A further integration of metabolome results and gene expression highlighted the regulation of metabolic changes and related pathways in severe cold exposure, such as “aminoacyl-tRNA biosynthesis” and “valine, leucine, and isoleucine degradation”. In summary, we presented new insights on the short-term effects of severe cold stress as well as metabolites and metabolic pathways associated with cold stress response in Inner-Mongolia Sanhe cattle.

**Abstract:**

Inner-Mongolia Sanhe cattle are well-adapted to low-temperature conditions, but the metabolic mechanisms underlying their climatic resilience are still unknown. Based on the ^1^H Nuclear Magnetic Resonance platform, 41 metabolites were identified and quantified in the serum of 10 heifers under thermal neutrality (5 °C), and subsequent exposure to hyper-cold temperature (−32 °C) for 3 h. Subsequently, 28 metabolites were pre-filtrated, and they provided better performance in multivariate analysis than that of using 41 metabolites. This indicated the need for pre-filtering of the metabolome data in a paired experimental design. In response to the cold exposure challenge, 19 metabolites associated with cold stress response were identified, mainly enriched in “aminoacyl-tRNA biosynthesis” and “valine, leucine, and isoleucine degradation”. A further integration of metabolome and gene expression highlighted the functional roles of the *DLD* (dihydrolipoamide dehydrogenase), *WARS* (tryptophanyl-tRNA synthetase), and *RARS* (arginyl-tRNA synthetase) genes in metabolic pathways of valine and leucine. Furthermore, the essential regulations of *SLC30A6* (solute carrier family 30 (zinc transporter), member 6) in metabolic transportation for propionate, acetate, valine, and leucine under severe cold exposure were observed. Our findings presented a comprehensive characterization of the serum metabolome of Inner-Mongolia Sanhe cattle, and contributed to a better understanding of the crucial roles of regulations in metabolites and metabolic pathways during cold stress events in cattle.

## 1. Introduction

Exposure to low environmental temperatures can severely affect growth [1], productive efficiency [2], reproductive performance [3], welfare [4], and immune response [5] of cattle. Therefore, low temperatures cause welfare and economic losses in the worldwide livestock industry, especially in northern countries [1,5,6,7]. Furthermore, according to a recent report from the Intergovernmental Panel on Climate Change (IPCC), the effects of climate change, including extreme weather events, are expected to further increase in the near future [8]. A better understanding of the biological mechanisms underlying thermal stress in livestock is paramount for developing mitigation strategies to minimize the negative effects of both heat and cold stress [9,10].

Inner-Mongolia Sanhe is a dual-purpose (milk and meat) cattle breed, which originated in the Inner-Mongolia Autonomous Region of China, where the winter is harsh and lasts for approximately 200 days a year [11]. Several studies have reported the high disease and cold stress resilience of Sanhe cattle in comparison to other taurine breeds [12,13,14]. We have also published associations between genomic polymorphisms and cold stress-related blood biochemical parameters [14], and the impact of cold stress on differential gene expression profiling in Sanhe cattle [11]. Therefore, Sanhe is a great resource for investigating the genetic background of cold stress response in cattle through the integration of multi-omics datasets.

Metabolites are the end product of various regulatory processes in the animal body, and fluctuations in their levels are the ultimate response of biological systems to the environmental challenges [15]. In this context, metabolomics has been shown to be a powerful tool for various fields, such as disease diagnosis [16], drug screening [17], food industry [18], and agriculture [19]. A study investigating *Staphylococcus aureus* response to prolonged exposure to cold stress revealed that citric acid and certain amino acids were involved in the rapid adaptation to low temperatures, indicating the importance of thermal homeostasis [20]. Metabolomic studies have also been employed to better understand coping mechanisms of cold tolerance in flies, in which remarkable metabolites and metabolic pathways related to cold tolerance were found [21,22]. In rats, the effects of both acute and chronic cold stress on their body fluids were investigated through metabolomics profiling and revealed important biochemical responses (e.g., tricarboxylic acid cycle, gut microbiota) to cold stress [23,24]. However, to the best of our knowledge, there are few studies reporting metabolomic profiling and biological mechanisms associated with cold-induced responses in livestock, especially in cattle.

Therefore, the main objectives of this study were to: (1) characterize the global metabolic profiling in the blood serum of Inner-Mongolia Sanhe cattle exposed to severe cold stress, and (2) determine the effects of cold stress on their metabolome and metabolic pathways. The findings of this study contribute to a better understanding of the biological processes and mechanisms underlying cold stress response in cattle and the definition of novel traits that can be used for genetic and genomic selection for improved climatic resilience in cattle.

## 2. Materials and Methods

### 2.1. Animals and Sample Collection

**Figure 1:**
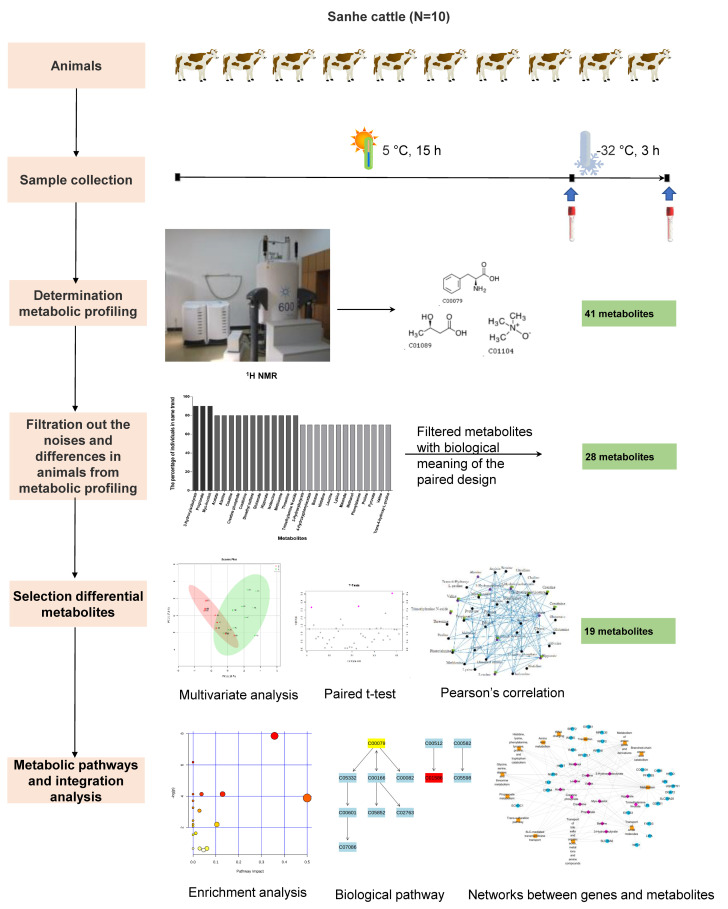
The analytical workflow performed in this study.

**Figure 2:**
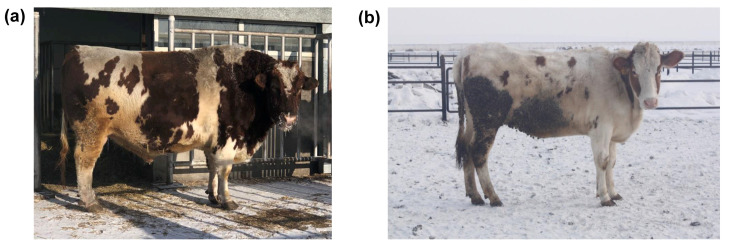
Sanhe cattle during severe cold stress exposure. (**a**) Young bull, and (**b**) Heifer.

Animal care was followed in agreement with the Committee on Ethics of Animal Experimentation from the Beijing Jiaotong University (Beijing, China) (ID: SS-QX-2014-06), and the experiment was performed according to regulations and guidelines established by this committee. Considering pedigree information and physiological condition that may cause the metabolites’ variation among individuals, 10 healthy and unrelated Inner-Mongolian Sanhe heifers with similar weight (430.0 ± 16.5 kg) and age (20.6 ± 1.3 months) were selected from the Xiertala Cattle Breeding Farm (Inner Mongolia, China). The heifers were semi-housed in the same cowshed and fed three times a day (5:00 a.m., 11:00 a.m., and 5:00 p.m.) with total mixed ration (TMR). Water was provided ad libitum during the whole experiment. In order to induce severe cold stress, the animals were transferred outdoors and exposed to a hyper-cold temperature (also mentioned as severe cold stress in the following text) of −32 °C for 3 h, followed by cowshed housing at 5 °C for 15 h (Figure 1, Figure 2b). The experiment was conducted during the winter season (January) and the environmental temperatures were measured by a handheld temperature equipment (with precision of 0.1 °C). The experimental procedures were conducted under the management of a local commercial farm in adverse winter conditions [13]. Non-anticoagulant blood samples (10 mL) were collected from the tail vein of each animal before and after exposure to cold stress, and then centrifuged at 1400× *g* for 10 min to obtain the upper serum. All serum samples were stored at −80 °C until subsequent analyses.

### 2.2. ^1^H Nuclear Magnetic Resonance (^1^H NMR) Analyses

In order to perform ^1^H Nuclear Magnetic Resonance (^1^H NMR), 20 serum samples (*n* = 10 before and *n* = 10 after exposure to severe cold stress; paired samples in 10 animals) were pre-treated and prepared as previously described by Beckonert et al. [25]. In brief, samples were centrifuged at 14,500× *g* for 15 min and the upper layer was transferred to a 0.5 mL 3 KDa ultrafiltration filter (Merck & Co., Inc, Kenilworth, NJ, USA). Samples were further centrifugated at 14,500× *g* for 45 min and a 450 μL aqueous layer was collected into a clean 2 mL centrifuge tube, followed by adding 50 μL of 2,2-dimethyl-2-silapentane-5-sulfonate (DSS) standard solution (Anachro Technologies Inc., Calgary, AB, Canada). Finally, the mixture was transferred to a 5 mm NMR tube. According to the instructions of the ^1^H NMR spectroscopy protocol, spectra were obtained using an Agilent DD2 600 MHz spectrometer equipped with a triple-resonance cryoprobe. The first increment of a 2D-1H, ^1^H-NOESY pulse sequence was utilized for the acquisition of ^1^H NMR data and suppressing the solvent signal. Here, 100 milliseconds mixing time along with 990 milliseconds pre-saturation (~80 Hz gammaB1) were used, and spectra data were collected at 25 °C, with a total of 128 scans over a period of 15 min. Finally, spectra data for Sanhe cattle serum were obtained.

The processing module in the Chenomx NMR Suite 8.1 software (Chenomx Inc., Edmonton, AB, Canada) was used to perform the automatically zero-filled and Fourier transform of the collected Free Induction Decay (FID) signal. The data was carefully phased, and the baseline was corrected in the Chenomx Processor. All the spectra were referenced to the internal standard and analyzed by experienced analysts against the Chenomx Compound Library. Lastly, the concentration information of all metabolites was normalized by weight across parallel samples prior to performing the multivariate analyses.

### 2.3. Metabolome Analysis

A subset of the original data was generated by retaining the metabolites with more than 70% of the samples in the same trend of change before and after the cold stress treatment (Figure 1). The MetaboAnalyst 4.0 software (www.metaboanalyst.ca/faces/home.xhtml, accessed on 25 January 2021) [26] was used to carry out the Principal Component Analysis (PCA) for investigating the clustering trends and outliers. Partial Least Squares Discriminant Analysis (PLS-DA) was performed to identify differential metabolites between before and after the cold stress exposure. The model quality of PLS-DA was determined by cross-validation (10-fold cross-validation, 9 groups for the training sets and 1 for the test set in each validation) based on the R^2^Y and Q^2^Y parameters, and 1000 random permutation tests to avoid model overfitting [19,27]. Furthermore, metabolites with Variable Importance in the Projection (VIP) values greater than 1 were considered as the most powerful group discriminators between before and after cold stress treatment, and reported as differential metabolites. A paired *t*-test was also used to select differential metabolites between before and after cold stress treatment considering a significance threshold of *p*-value < 0.05. The Pearson correlations of each two metabolites before and after cold stress exposure were calculated using the “Hmisc” package implemented in the R software (v 3.3.2) [28]. The Edraw Mind Map software (Edraw Software Co., Ltd., Shenzhen, Guangdong, China) was used to perform the visualization of metabolite correlations with *p*-value < 0.05. All differential metabolites were analyzed using MetaboAnalyst 4.0 (www.metaboanalyst.ca/faces/home.xhtml, accessed on 26 January 2021) through the Metabolite Set Enrichment Analysis (MSEA).

### 2.4. Integration Analysis of Transcriptome and Metabolome Datasets

To further illustrate the differential metabolites and their related pathways, an integration analysis was performed based on a published dataset of expression microarray in blood samples [11]. This dataset was derived from three individuals, before and after exposure to severe cold stress, which belonged to the same population of metabolome data in this current study. A total of 193 genes were differentially expressed with significant biological changes (fold change ≥ 1.3 or *p* < 0.05), and then integrated with the metabolome data. The MetaboAnalyst 4.0 package (www.metaboanalyst.ca/faces/home.xhtml, accessed on 29 January 2021) with a list of gene and compound names as input was used to identify candidate genes involved in key metabolic pathways. In addition, the IMPaLA online software (http://impala.molgen.mpg.de./, accessed on 29 January 2021) was used to integrate genes and metabolites into pathways corresponding to relevant biological processes [29]. The visualization of networks between genes and metabolites was performed using the Cytoscape software (v3.7.2, US National Institute of General Medical Sciences, Bethesda, MD, USA) [30].

## 3. Results

### 3.1. The Serum Metabolome in Inner-Mongolia Sanhe Cattle

**Figure 3:**
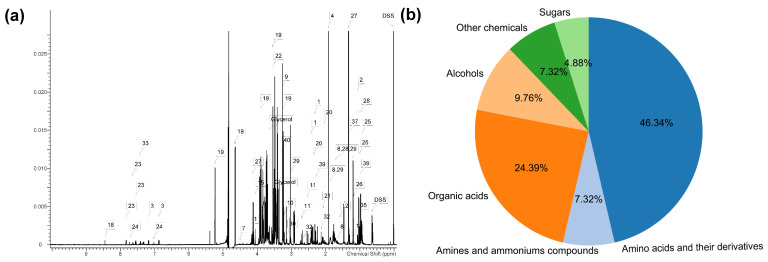
The serum metabolic profiling of Sanhe cattle. (**a**) The ^1^H NMR spectra (0–10.0 ppm) of serum from Sanhe cattle. The numbers represent signals of equivalent hydrogen, and the DSS is 0 ppm for the chemical shift of the whole spectrum. (**b**) Forty-one unique metabolites were commonly identified and quantified in the serum metabolic profiling of Sanhe cattle.

A set of 41 metabolites were commonly identified and quantified with 600 MHz ^1^H NMR (0–10 ppm) spectra in the serum metabolic profiling of Sanhe cattle (Figure 3a), which corresponds to an average of 40 compounds per sample. These metabolites included 19 amino acids and their derivatives (alanine, arginine, betaine, citrulline, creatine, glutamate, glutamine, glycine, hippurate, histidine, isoleucine, leucine, lysine, methionine, phenylalanine, proline, threonine, trans-4-hydroxy-l-proline, and valine), 3 amine and ammonium compounds (creatinine, choline, and trimethylamine N-oxide), 10 organic acids (3-hydroxybutyrate, 3-hydroxyisobutyrate, 4-hydroxyphenylacetate, acetate, citrate, formate, lactate, malonate, propionate, and pyruvate), 4 alcohols (ethanol, isopropanol, methanol, and myo-Inositol), 2 sugars (arabinose, and glucose), and 3 other chemicals (dimethyl sulfone, acetone, and creatine phosphate) (Figure 3b). As shown in Table 1, the metabolites in the serum of Sanhe cattle were involved in the metabolism of amino acids, carbohydrates, and lipids, as well as gut microbiome-derived metabolism. On the other hand, the identified metabolites presented different abundance in the Sanhe cattle serum, and the three most abundant metabolites found in the serum were glucose (with a mean value of 3.394 mmol/L), followed by acetate (0.704 mmol/L), and 3-hydroxybutyrate (0.274 mmol/L), while the three least detectable metabolites were isopropanol (0.0045 mmol/L), choline (0.0095 mmol/L), and creatine phosphate (0.010 mmol/L). Supplementary Table S1 contains the complete set of the 41 confirmed compounds in Sanhe cattle serum and their concentrations.

### 3.2. Pre-Selection of Candidate Metabolites Related to Severe Cold Stress in Sanhe Cattle

**Figure 4:**
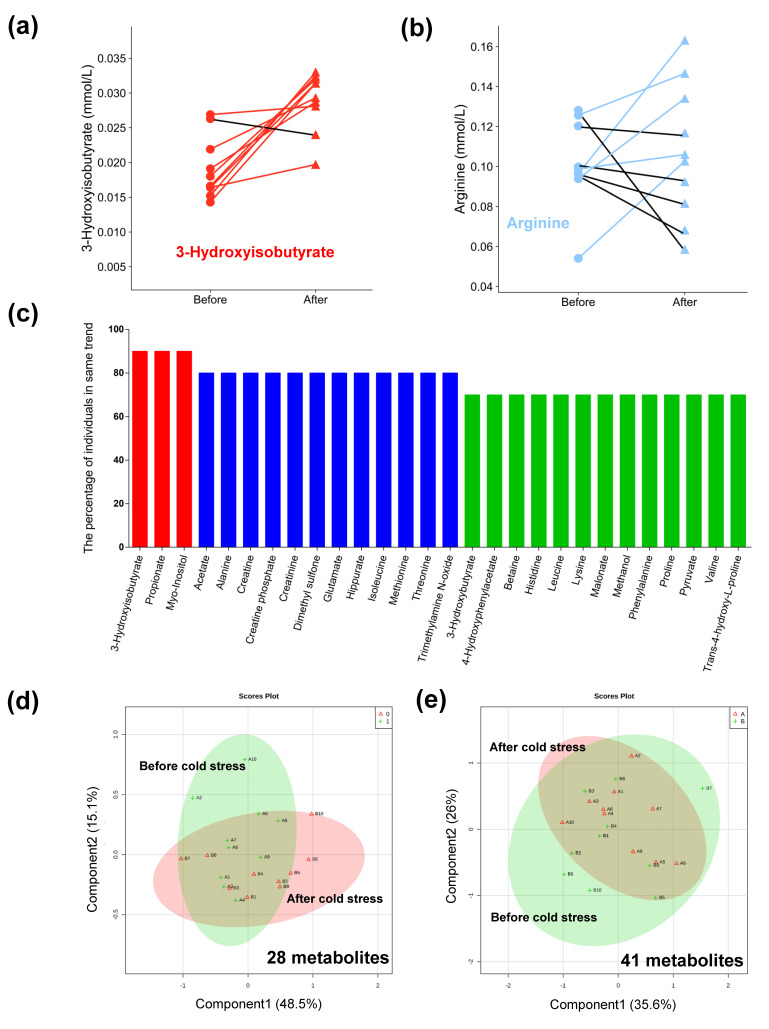
The pre-selection of metabolic profiling. (**a**) The changes of 3-hydroxyisobutyrate in 10 animals exposed to severe cold. (**b**) The changes of arginine in 10 animals exposed to severe cold. (**c**) Twenty-eight metabolites changed in the same trend after cold stress. (**d**) Score plots of PCA with 28 metabolites (R^2^X = 0.636). (**e**) Score plots of PCA with 41 metabolites (R^2^X = 0.616).

In order to evaluate the metabolic response to severe cold stress in 10 experimental animals, comparison between the two concentration values of each metabolite corresponding to before and after cold stress was conducted for each animal. As shown in Figure 4a,b, the metabolic changes varied in 10 experimental animals under cold stress exposure. For instance, the concentration value of 3-hydroxyisobutyrate increased in nine animals exposed to severe cold stress, while arginine increased in five heifers but decreased in the other five individuals. It revealed individual variability in metabolic changes when animals experienced cold stress. Moreover, those irregular metabolites may either be the noises from the baseline during detection or show irrelevant variables that will not be influenced by cold stress. To find the candidate metabolites altered by severe cold stress instead of individual variation and detection noises, a pre-selection analysis of all metabolites was performed based on whether they had similar population patterns after severe cold exposure. In the present study, if one metabolite increased or decreased in ≥70% of animals after cold exposure, this metabolic change was considered to be induced by cold stress. Filtering the raw metabolic data with the above biological significance, 28 out of the 41 metabolites remained for further analyses, and Figure 4c indicates good agreement in the changed signs of 28 candidate metabolites in the 10 individuals. The score plot based on PCA with 28 metabolites displays a clear separation between the two groups (before and after cold exposure) compared to the profiling of 41 metabolites (Figure 4d,e).

### 3.3. Metabolite Changes in the Serum of Sanhe Cattle after Severe Cold Stress

**Figure 5:**
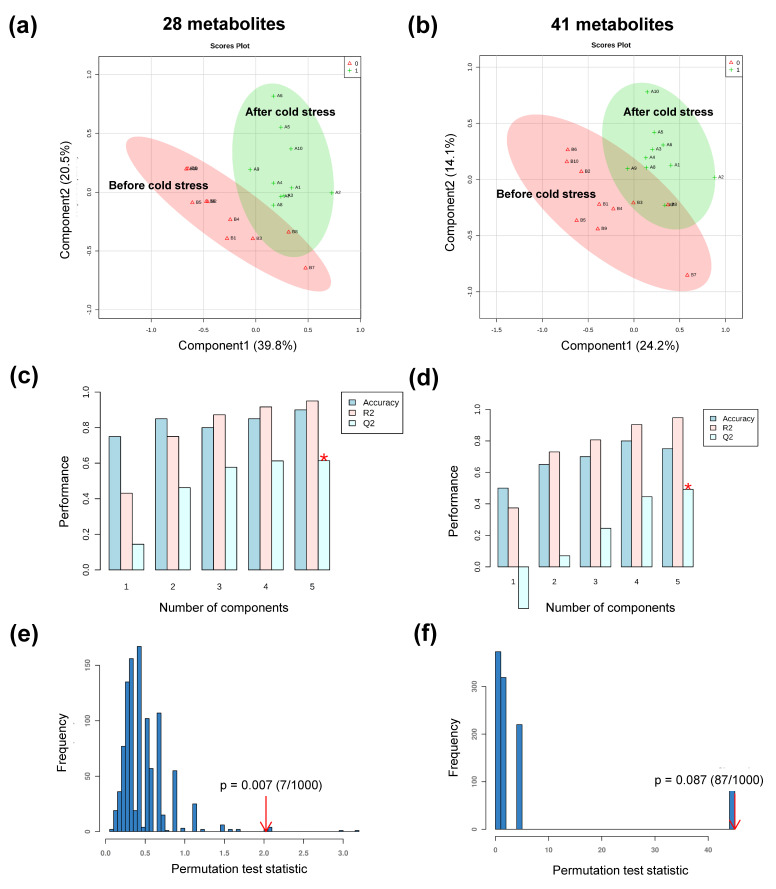
PLS-DA analyses of metabolic profiling with 28 and 41 metabolites. (**a**) Score plots of PLS-DA with 28 metabolites (R^2^X = 0.603). (**b**) Score plots of PLS-DA with 41 metabolites (R^2^X = 0.383). (**c**) Cross-validation results of PLS-DA with 28 metabolites (components = 5, R^2^Y = 0.95, Q^2^Y = 0.62). (**d**) Cross-validation results of PLS-DA with 41 metabolites (components = 5, R^2^Y = 0.94, Q^2^Y = 0.49). (**e**) 1000 random permutation tests in PLS-DA with 28 metabolites (*p* = 0.007). (**f**) 1000 random permutation tests in PLS-DA with 41 metabolites (*p* = 0.087). *: *p*-Value < 0.05.

As shown in Figure 5, PLS-DA was used to achieve the optimum distinction between the pre- and post-cold stress groups and classify the differential metabolites. The first two components explained 60.3% of the total variance in X (R^2^X = 0.603) and displayed a strong distinction between the two groups, with a high total variance of Y (Figure 5a, R^2^Y = 0.95) and a good predictability in cross-validation (Figure 5c, Q^2^Y = 0.62). Furthermore, the validation with 1000 random permutation tests provided *p* = 0.007, indicating a low probability of overfitting in the model used (Figure 5e). At the same time, PLS-DA was also used to check the raw data of 41 metabolites (Figure 5b,d,f). The multivariate analysis of the 28 metabolites showed a clear separation trend, not only based on PCA but also on PLS-DA, with higher R^2^Y and Q^2^Y values as well as lower *p*-values in the permutation test. Hence, the current method could be an effective approach for excluding the irrelevant metabolites corresponding to individual variation and baseline noises from the raw metabolome. Furthermore, these 28 important metabolites were used to conduct paired Student’s *t*-tests and Pearson correlation analysis.

The VIP value of each metabolite within the PLS-DA model is typically thought to be potent for group discriminators. Firstly, the VIP values were calculated to indicate their contribution to classification, and VIP > 1 was used as a threshold to filter the significantly changed metabolites after acute cold stress. Nine metabolites were found to account for variations between cold stress and the control group, and the top two metabolites were 3-hydroxybutyrate (VIP = 2.44) and trimethylamine N-oxide (VIP = 2.07). The detailed information of these nine metabolites is shown in Table 2. Secondly, based on the paired Student’s *t*-test at the univariate level, the concentrations of 12 metabolites significantly increased under cold stress (*p* < 0.05), and the trimethylamine N-oxide had the lowest *p*-value (0.001). Taken together, six metabolites (trimethylamine N-oxide, methanol, hippurate, valine, 3-hydroxyisobutyrate, and leucine) were found to have both VIP value > 1 and *p*-value < 0.05 (Table 2).

### 3.4. Changes of Correlation between Candidate Metabolites under Severe Cold Stress

**Figure 6:**
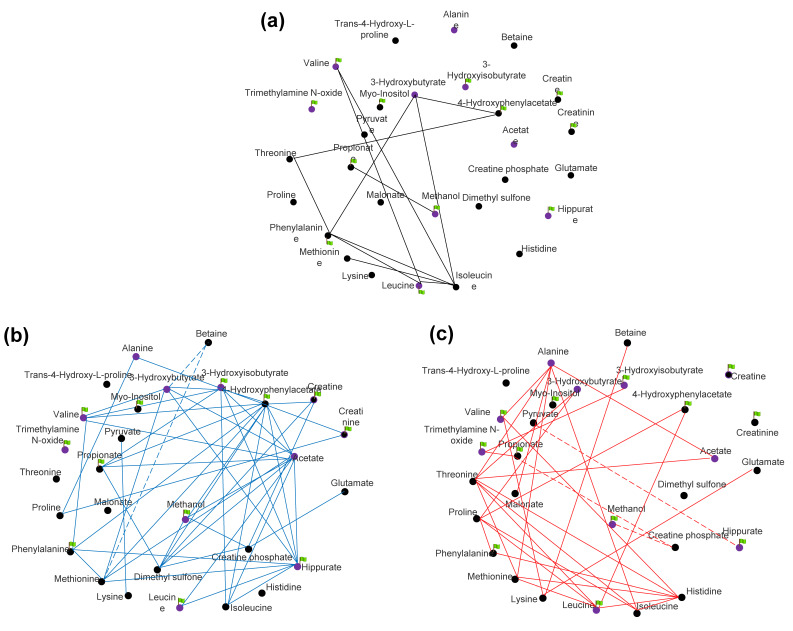
Correlation-based networks of 28 metabolites in Sanhe cattle. (**a**) The correlation among metabolites that were common in the serum of Sanhe cattle before and after severe cold stress. (**b**) The correlation among metabolites that were only found in the serum of Sanhe cattle before severe cold stress. (**c**) The correlation among metabolites that were only found in the serum of Sanhe cattle after severe cold stress. The solid line is significantly positive correlated, while the dotted line is significantly negative correlated. The purple circles are the metabolites with a VIP value > 1 in PLS-DA, and the green flags are the metabolites with a *p*-value < 0.05 in the paired *t*-test.

A total of 28 metabolites under two conditions were used to make the metabolic profiles and to be acquired for correlation analysis. Under normal conditions, 57 pairs of metabolites showed a significant correlation (55 positive and two negative correlations, Figure 6a,b), while after cold stress exposure, 50 pairs were found to be significantly correlated (47 positive and three negative correlations, Figure 6a,c), with only 12 out of 107 metabolite pairs showing no alteration after cold stress exposure (Figure 6a). Based on the Pearson’s correlation coefficient of each two metabolites generated from before and after cold stress, a total of 12 metabolite pairs were determined with the difference of correlation coefficient being more than 1 between before and after cold exposure (Supplementary Table S2). Highly significant changes of correlation were observed in creatine phosphate and methanol, which showed a strong positive correlation in neutral temperature (r = 0.78, *p* < 0.01), but highly negative correlations were seen after cold stress (r = −0.82, *p* < 0.01). Overall, 12 metabolites were identified to be discrepant metabolites (Table 2).

### 3.5. Key Metabolic Pathways Involved in Severe Cold Stress

**Figure 7:**
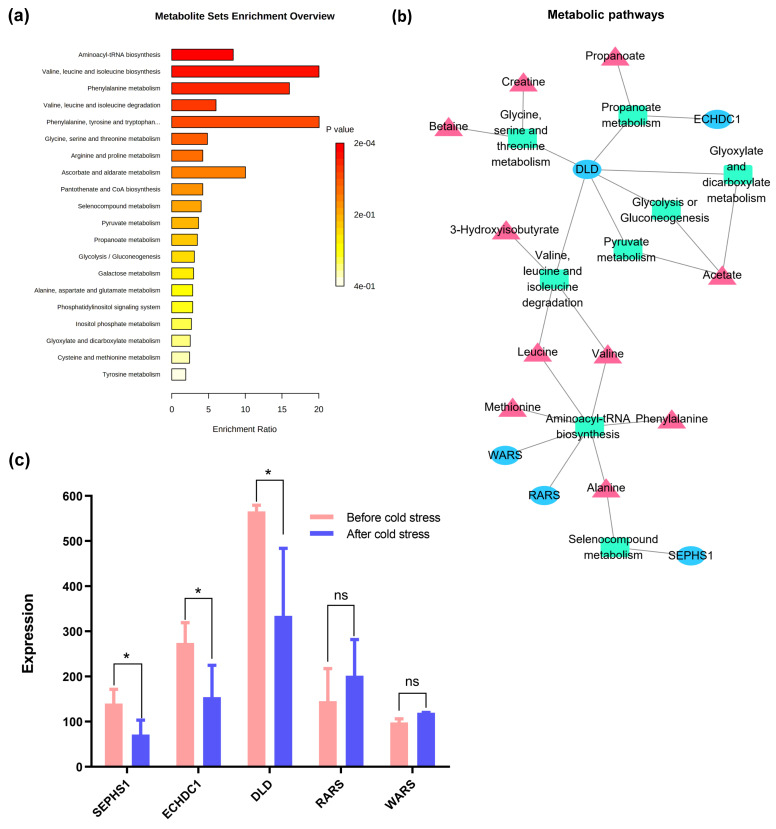
Networks between metabolites and genes involved in metabolic pathways. (**a**) Overview for metabolite set enrichment analysis of metabolic response to cold stress in Sanhe cattle. (**b**) Relevant metabolic pathways involved in the response to cold stress in Sanhe cattle. Triangles represent metabolites, ovals represent genes, and rectangles are the metabolic pathways. (**c**) The changes of gene expressions involved in metabolic pathways. *: *p*-Value < 0.05; ns: no significance; *SEPHS1*: selenophosphate synthetase 1; *ECHDC1*: enoyl coenzyme A hydratase domain containing 1; *DLD*: dihydrolipoamide dehydrogenase; *RARS*: arginyl-tRNA synthetase; *WARS*: tryptophanyl-tRNA synthetase.

Based on the thresholds of VIP > 1 in PLS-DA, *p*-value < 0.05 in the paired *t*-test, or metabolites pairs with the difference in correlation coefficient > 1, a total of 19 metabolites in the serum of Sanhe cattle were identified to be significantly associated with cold stress (Table 2). A total of 20 metabolic pathways were found to be enriched based on the MSEA analysis (Supplementary Table S3). As shown in Figure 7a, “aminoacyl-tRNA biosynthesis” and “valine, leucine, and isoleucine degradation” were the top two enriched pathways, with five and three metabolites, respectively. In addition, “phenylalanine metabolism”, “pyruvate metabolism”, “propanoate metabolism”, and “glycolysis/gluconeogenesis” were identified and are mainly related to five metabolites (propionate, acetate, phenylalanine, valine, and leucine).

We then performed the integration analysis based on the datasets of 19 differential metabolites and 193 candidate genes from our previous study [9], with detailed results provided in Supplementary Table S4. Eight metabolic pathways were identified (Figure 7b), in which five genes, including three downregulated (selenophosphate synthetase 1, *SEPHS1*; enoyl coenzyme A hydratase domain containing 1, *ECHDC1*; dihydrolipoamide dehydrogenase, *DLD*) and two upregulated (arginyl-tRNA synthetase, *RARS*; tryptophanyl-tRNA synthetase, *WARS*) genes (Figure 7c) were found to play functional roles in the metabolic pathways of ten related metabolites (propionate, acetate, phenylalanine, valine, leucine, creatine, betaine, 3-hydroxyisobutyrate, methionine, and alanine). Both *DLD* and acetate were observed to be involved in three pathways, e.g., “glyoxylate and dicarboxylate metabolism”, “glycolysis/gluconeogenesis”, and “pyruvate metabolism”. Two pathways, “valine, leucine, and isoleucine degradation” and “aminoacyl-tRNA biosynthesis”, were commonly enriched with three genes (*DLD*, *WARS*, and *RARS*) and two metabolites (valine and leucine). These findings indicated that cold stress induced metabolic changes and activated related metabolic pathways.

### 3.6. Significant Biological Processes Related to Metabolites’ Regulation in Severe Cold Stress

**Figure 8:**
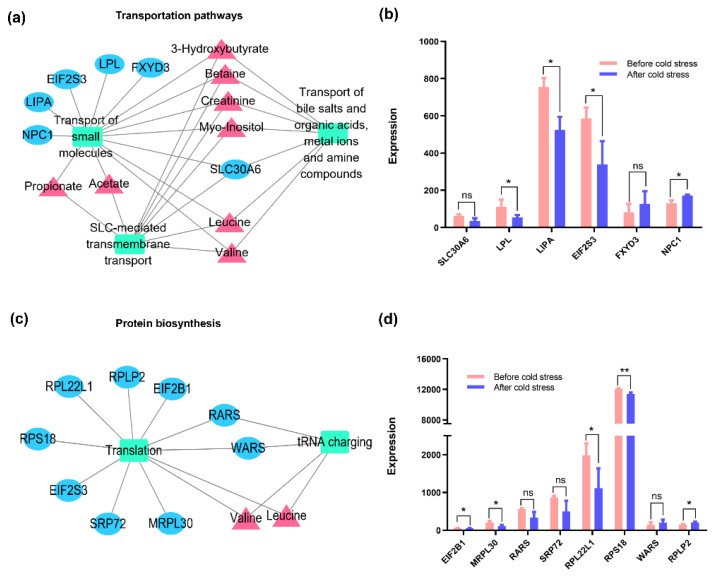
Networks between metabolites and genes involved in transportation pathways and protein biosynthesis. (**a**) Transportation pathways. (**b**) Protein biosynthesis. Triangles represent metabolites, ovals represent genes, and rectangles represent the metabolic pathways. (**c**) The changes of gene expressions involved in transportation pathways. (**d**) The changes of gene expressions involved in protein biosynthesis. *: *p*-Value < 0.05; **: *p*-Value < 0.01; ns: no significance; *SLC30A6*: solute carrier family 30 (zinc transporter), member 6; *LPL*: lipoprotein lipase; *LIPA*: lipase A, lysosomal acid, cholesterol esterase; *EIF2S3*: eukaryotic translation initiation factor 2, subunit 3 gamma; *FXYD3*: FXYD domain containing ion transport regulator 3; *NPC1*: Niemann-Pick disease, type C1; *EIF2B1*: eukaryotic translation initiation factor 2B, subunit 1 alpha; *MRPL30*: mitochondrial ribosomal protein L30; *RARS*: arginyl-tRNA synthetase; *SRP72*: signal recognition particle 72 kDa; *RPL22L1*: ribosomal protein L22-like 1; *RPS18*: ribosomal protein S18; *WARS*: tryptophanyl-tRNA synthetase; *RPLP2*: ribosomal protein, large, P2.

**Figure 9:**
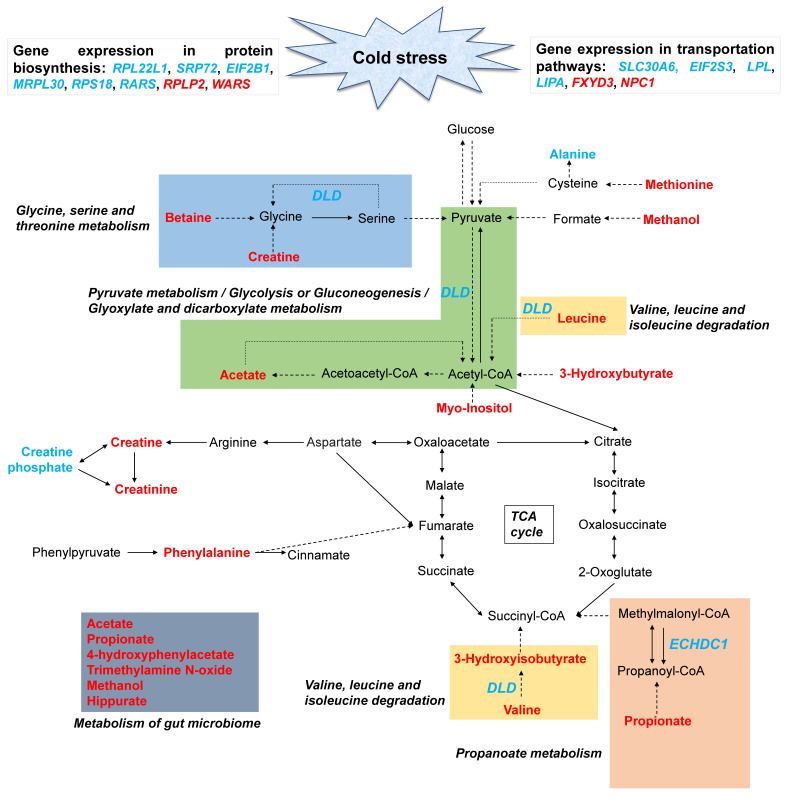
Proposed mechanisms of cold stress response to related functional pathways. Red indicates upregulated metabolites, and blue represents downregulated metabolites, however, blue italics indicates downregulated genes. *RPL22L1*: ribosomal protein L22-like 1; *SRP72*: signal recognition particle 72 kDa; *EIF2B1*: eukaryotic translation initiation factor 2B, subunit 1 alpha; *MRPL30*: mitochondrial ribosomal protein L30; *RPS18*: ribosomal protein S18; *RARS*: arginyl-tRNA synthetase; *RPLP2*: ribosomal protein, large, P2; *WARS*: tryptophanyl-tRNA synthetase; *SLC30A6*: solute carrier family 30 (zinc transporter), member 6; *EIF2S3*: eukaryotic translation initiation factor 2, subunit 3 gamma; *LPL*: lipoprotein lipase; *LIPA*: lipase A, lysosomal acid, cholesterol esterase; *FXYD3*: FXYD domain containing ion transport regulator 3; *NPC1*: Niemann-Pick disease, type C1; *DLD*: dihydrolipoamide dehydrogenase; *ECHDC1*: enoyl coenzyme A hydratase domain containing 1.

Figure 8a,b highlight the associated pathways in metabolic transportation, such as “SLC-mediated transmembrane transport”, “transport of bile salts and organic acids”, “metal ions and amine compounds”, and “transport of small molecules”. Among them, one (solute carrier family 30 (zinc transporter), member 6, *SLC30A6*) out of six genes were commonly linked to these pathways and related to eight metabolites (such as propionate, acetate, valine, and leucine), suggesting the perspective of changeable transportation efficiency for most metabolites when exposed to cold stress. Additionally, valine and leucine were identified to be enriched in translation and tRNA charging (Figure 8c,d), and eight genes were differentially expressed in those relevant processes which could further discriminate the effects of cold stress on protein biosynthesis (Figure 9).

## 4. Discussion

Inner-Mongolia Sanhe is a dual-purpose cattle breed in China that has been an important genetic resource for studying the effects of cold stress response on blood biochemical parameters [31], gene expression profile in the peripheral blood [9], characterization of genetic variation related to cold tolerance [14], and signatures of selection related to thermal tolerance [32]. However, serum metabolome and metabolic regulation in response to severe cold exposure had not been studied in this population. Considering the balance between analytical cost and statistical power, 10 Sanhe heifers were used in this experiment. Forty-one unique metabolites were identified, and to the best of our knowledge, this is the first characterization of the metabolic profiling of serum in this local breed [19]. Some of the most abundant molecules are involved in carbohydrate metabolism, while the least abundant compounds serve as intermediate products in the metabolism of amino acids and lipids. Furthermore, this metabolome data also provides basic information of serum compounds in cattle. 

### 4.1. The Pre-Filtration for the Paired Metabolome Data

The paired experimental design, which differs from the ordinary case-control design with its feature of the pairs of observations, was conducted for identifying differential metabolites in our study. This approach has been widely used in clinical and behavioral studies in humans [33,34] and livestock feeding trials [35,36]. In addition, as a typical design in which the baseline of all subjects is observed in pre-intervention (as control), it can increase the effectiveness of univariate tests. However, the challenge for multivariate analyses is that paired metabolome data is still not accommodated by the most common software, such as SIMCA-P (http://umetrics.com/products/simca, accessed on 20 December 2020) [37] and XCMS Online (https://xcmsonline.scripps.edu, accessed on 20 December 2020) [38]. MetaboAnalyst (www.metaboanalyst.ca/faces/home.xhtml, accessed on 20 December 2020) allows the users to upload a list of metabolite pairs [26], but the score and loading plots of PCA and PLS-DA for paired data are completely the same as the unpaired one (Supplementary Figure S1), neglecting the powerful biological interpretation of paired data. Most studies have analyzed the paired structure metabolome data under the classical case and control approach [39,40], and most importantly, the large amount of information contained in the pairing design were ignored. In our study, the pre-selection made a reform with more obvious separation trends in score plots of both PCA and PLS-DA, as well as higher R^2^Y and Q^2^Y of PLS-DA with values of 0.95% (0.95% versus 0.94%) and 0.62% (0.62% versus 0.49%, increased 26.5%) respectively, and a goodness of fit in PLS-DA with a lower *p*-value in 1000 permutation tests (0.007% versus 0.087%). In general, R^2^Y represents the cumulative interpretation ability, and Q^2^Y indicates the predictive ability, and the PLS-DA model can be accepted when they are both greater than 0.5 [19]. Besides, permutation tests are used to verify the probability of overfitting [27], and the lower *p*-value indicated a goodness of fit in the given PLS-DA model with 28 metabolites. The results suggest that the statistical power in multivariate analyses significantly improved by using the method of pre-filtration. Similarly, Westerhuis et al. [41] have proposed the consideration of individual variations with the method of multilevel PLS-DA for cross-over design. Thiébaut et al. [42] then described it with an extension for two-factor data. In our case, as the impact of cold exposure may differ among animals, the pre-filtration was performed according to the biological meaning, following previous studies [41,42]. Furthermore, options in pre-selection also removed the noises of the baseline from the detection. Overall, our findings provide strong evidence that the pre-selection for metabolites through the biological significance of paired design is an effective way to reduce the effects of other factors in the multivariate approach.

### 4.2. The Metabolic Pathways’ Response to Severe Cold Stress in Sanhe Cattle

Nineteen metabolites in the serum of Sanhe cattle significantly changed after the animals were exposed to severe cold stress. Generally, organic acids, amino acids, and their derivatives are distributed for 95% of all differential metabolites, which may account for the features of the ^1^H NMR method [43]. On the other hand, this study indicated that the changes of metabolite concentrations in serum following cold stress can be investigated using the ^1^H NMR platform. Moreover, 17 upregulated metabolites, such as 3-hydroxybutyrate, propionate, acetate, valine, and leucine, were observed when individuals experienced severe cold stress, while only two metabolites (alanine and creatine phosphate) were downregulated (Figure 9). These findings suggest that short-term exposure to severe cold stress induced the upregulation of energy supply, thereby resulting in elevated circulation of a number of important compounds. Furthermore, the gene expressions involved in metabolic pathways, transportation pathways, and protein biosynthesis were affected concomitantly. Furthermore, there was little overlap among the metabolites identified in this study when compared to the cold stress responses in the urine samples of rats under both acute and chronic cold stress patterns [23,25,44], in the liver of yellow drum [45], and in the blood of dairy goats [46] exposed to cold stress. These studies reveal the diversity of metabolic regulation under cold conditions due to the differences presented in experimental species, detection platform, and the degree and intensity of the cold treatments. 

In the current study, 3-hydroxybutyrate, as the representative metabolite with the largest VIP value, is a key ketone body from fat metabolism that supplies energy and is associated with the adaptive response to multiple sources of stress [47]. Previous studies reported a significant difference in the level of 3-hydroxybutyrate between control and heat-stressed animals, such as in the plasma and milk of dairy cows [37,48], serum of broilers [49], and serum of finishing pigs [50]. These results indicate a similar mechanism of metabolic compensatory regulation responding to cold and heat stress. Moreover, cold stress might contribute to the regulation of amino acids’ metabolism [23]. The metabolic pathways of “glycine, serine, and threonine metabolism” and “valine, leucine, and isoleucine degradation” were altered in the serum of Sanhe cattle in response to cold stress, which is in agreement with the increased concentration of involved compounds, such as betaine, creatine, 3-hydroxyisobutyrate, leucine, and valine. Valine and leucine are branched chain amino acids that can uptake into the tricarboxylic acid cycle to increase energy supply. Indeed, the relationship between their concentration and stress has been previously reported in rats under foot shock [51] and psychological stress [52]. Additionally, the serum levels of creatine and creatinine, which play important roles in energy balance when animals have high energy requirements [53], were higher after exposure to cold stress in our study. Therefore, cold stress induced the increased concentrations in those amino acids, and their intermediate products indicate that the metabolism of amino acids has been derived following a period of severe cold stress. At the same time, valine and leucine were also found to be enriched in the pathways of transportation and protein biosynthesis. However, the genes involved in those pathways were downregulated, suggesting that the utilization of valine and leucine may be inhibited in the leukocytes when Sanhe cattle experience severe cold conditions. Additionally, the stress-related *DLD* (dihydrolipoamide dehydrogenase) gene (Figure 9), which can generate the mitochondrial enzyme with diaphorase activity and associate with multiple pathways, was observed to be downregulated in circulating leukocytes. This evidence provides functional evidence for the above results. 

In the terms of acetate and propionate, they are the volatile fatty acids (VFA) from the metabolism of microorganisms in the rumen, and mostly attribute to generate glucose by gluconeogenesis [54,55]. Given the significantly different concentrations of the above two metabolites between cattle under thermoneutral conditions and after cold exposure, our results indicate that VFA might also play an important role in the regulation of cold response in Sanhe cattle. Not surprisingly, the expressions of most genes in transportation pathways (including “transport of small molecules” and “SLC-mediated transmembrane transport”) for acetate and propionate were decreased in the leukocytes after exposure to cold conditions. Among those crucial genes, *SLC30A6*, which is an important zinc transporter from the *SLC30* family, was shared in two transportation pathways and found to be involved in the cellular transportation of acetate and propionate. The corresponding lower expression in circulating blood may represent the metabolic adaptation mechanism. Furthermore, we also observed that cold exposure influenced the excretion levels of 4-hydroxyphenylacetate, trimethylamine N-oxide, methanol, and hippurate, all of which are considered to be involved in the metabolism of the gut microbiome [37,56]. These findings show the involvement of gut microbiota in the response to cold exposure in Sanhe cattle. However, the regulation mechanisms should be studied further.

## 5. Conclusions

There were 41 metabolites identified and quantified in the serum metabolic profiling of Inner-Mongolia Sanhe cattle using the ^1^H NMR platform, and 28 metabolites were found as potential cold-related variations by the biological significance of paired design. By combining further analyses of PLS-DA, paired *t*-test, and correlation network, 19 metabolites were determined to be differential metabolites and involved in the metabolic regulation of fat metabolism, amino acid metabolism, and gut microbial metabolism to cold response in Sanhe cattle. The results of integration analysis with transcriptome and metabolome data further clarified that cold stress induced metabolic changes and activated related metabolic pathways. Our results provide novel insights on the shifts in metabolic pathways for energy supply on the responses to cold stress in cattle.

## Figures and Tables

**Table 1:** Forty-one metabolites and their metabolic pathways in the serum of Inner-Mongolia Sanhe cattle.

No.	Metabolite	Metabolic Pathway	No.	Metabolite	Metabolic Pathway
1	3-Hydrxyisobutyrate	Amino acid	22	Trans-4-Hydroxy-l-proline	Amino acid
2	4-Hydroxyphenylacetate	Amino acid	23	Betaine	Betaine metabolism
3	Alanine	Amino acid	24	Acetate	Carbohydrate
4	Arginine	Amino acid	25	Arabinose	Carbohydrate
5	Citrulline	Amino acid	26	Citrate	Carbohydrate
6	Creatine	Amino acid	27	Ethanol	Carbohydrate
7	Creatine phosphate	Amino acid	28	Formate	Carbohydrate
8	Creatinine	Amino acid	29	Glucose	Carbohydrate
9	Glutamate	Amino acid	30	Isopropanol	Carbohydrate
10	Glutamine	Amino acid	31	Lactate	Carbohydrate
11	Glycine	Amino acid	32	Methanol	Carbohydrate
12	Hippurate	Amino acid	33	Propionate	Carbohydrate
13	Histidine	Amino acid	34	Pyruvate	Carbohydrate
14	Isoleucine	Amino acid	35	Myo-Inositol	Carbohydrate
15	Leucine	Amino acid	36	Trimethylamine N-oxide	Gut microbiome-derived metabolism
16	Lysine	Amino acid	37	3-Hydroxybutyrate	Lipid
17	Methionine	Amino acid	38	Acetone	Lipid
18	Phenylalanine	Amino acid	39	Choline	Lipid
19	Proline	Amino acid	40	Malonate	Lipid
20	Threonine	Amino acid	41	Dimethyl sulfone	Sulfur metabolism
21	Valine	Amino acid			

**Table 2:** Metabolites changed in serum of Sanhe cattle after acute cold stress.

Metabolite	VIP ^1^	*p*-Value ^2^	Correlation Coefficient Difference ^3^	Regulation Status ^4^
3-Hydroxybutyrate	2.44	0.072	>1	up
Trimethylamine N-oxide	2.07	0.001	NA	up
Methanol	1.43	0.050	>1	up
Hippurate	1.34	0.002	>1	up
Acetate	1.33	0.400	NA	up
Valine	1.29	0.050	>1	up
3-Hydroxyisobutyrate	1.22	0.002	>1	up
Alanine	1.08	NA	NA	down
Leucine	1.06	0.050	NA	up
4-Hydroxyphenylacetate	0.84	0.027	>1	up
Betaine	0.06	0.830	>1	up
Creatine	0.92	0.049	>1	up
Creatine phosphate	0.44	0.086	>1	down
Creatinine	0.85	0.010	NA	up
Dimethyl sulfone	0.31	0.170	>1	up
Methionine	0.43	NA	>1	up
Myo-Inositol	0.62	0.050	NA	up
Phenylalanine	0.81	0.050	NA	up
Propionate	0.79	0.011	>1	up

^1^ Variable Importance in the Projection (VIP) values in Partial Least Squares-Discriminant Analysis (PLS-DA). ^2^ *p*-Value in paired *t*-test. ^3^ The difference of correlation coefficients was more than 1. ^4^ The upregulated means the higher concentration in the serum after cold stress. NA: not applicable.

## Data Availability

All the relevant data are provided in the main text or the Supplementary Materials.

## Supplementary Materials

The following are available online at https://www.mdpi.com/article/10.3390/ani11092493/s1, Figure S1: The comparison between standard PLS-DA and paired PLS-DA with 41 metabolites, Table S1: The concentrations of metabolites in the serum of Sanhe cattle, Table S2: Metabolite pairs with a difference of correlation coefficient of more than 1 between before and after severe cold stress in Sanhe cattle, Table S3: Relevant metabolic pathway by enrichment analysis with 19 differential metabolites in Sanhe cattle, Table S4: Networks between metabolites and genes associated with severe cold stress.
