# Supplementary material for: Investigating the Short-Term Effects of Cold Stress on Metabolite Responses and Metabolic Pathways in Inner-Mongolia Sanhe Cattle
Source: Animals (Basel). 2021 Aug 25;11(9):2493. doi: 10.3390/ani11092493 (PMC8469163; doi:10.3390/ani11092493)
Supplement: Supplementary file 1 [file animals-11-02493-s001.zip › animals-1316094-supplementary.pdf]

Table S1. The concentrations of metabolites in the serum of Sanhe cattle.

	Before (mmol/L)		After (mmol/L)	
Metabolites	Range	Mean	Range	Mean
3-Hydroxybutyrate	0.132-0.364	0.242	0.268-0.371	0.305
3-Hydroxyisobutyrate	0.014-0.027	0.019	0.020-0.033	0.029
4-Hydroxyphenylacetate	0.024-0.045	0.033	0.027-0.051	0.040
Acetate	0.346-1.126	0.675	0.511-0.889	0.732
Acetone	0.010-0.029	0.016	0.015-0.021	0.018
Alanine	0.145-0.232	0.183	0.098-0.242	0.163
Arabinose	0.016-0.028	0.020	0.000-0.034	0.017
Arginine	0.054-0.128	0.101	0.059-0.163	0.107
Betaine	0.108-0.24	0.160	0.096-0.276	0.159
Choline	0.008-0.012	0.010	0.008-0.012	0.009
Citrate	0.119-0.231	0.161	0.114-0.224	0.164
Citrulline	0.033-0.083	0.050	0.033-0.086	0.057
Creatine	0.150-0.221	0.181	0.154-0.242	0.195
Creatine phosphate	0.008-0.016	0.012	0.000-0.021	0.008
Creatinine	0.053-0.109	0.080	0.062-0.108	0.091
Dimethyl sulfone	0.013-0.052	0.024	0.015-0.043	0.027
Ethanol	0.010-0.016	0.013	0.008-0.018	0.013
Formate	0.021-0.04	0.027	0.021-0.036	0.027
Glucose	3.190-3.665	3.372	2.987-3.785	3.416
Glutamate	0.028-0.082	0.049	0.032-0.07	0.049
Glutamine	0.102-0.189	0.143	0.118-0.187	0.151
Glycine	0.077-0.17	0.118	0.070-0.204	0.119
Hippurate	0.021-0.038	0.031	0.033-0.053	0.044
Histidine	0.035-0.047	0.041	0.026-0.047	0.038
Isoleucine	0.063-0.104	0.081	0.063-0.128	0.091
Isopropanol	0.003-0.005	0.004	0.003-0.007	0.005
Lactate	0.406-1.592	0.846	0.423-1.348	0.853
Leucine	0.075-0.136	0.102	0.087-0.148	0.118
Lysine	0.058-0.078	0.068	0.041-0.108	0.076
Malonate	0.010-0.021	0.015	0.013-0.022	0.017
Methanol	0.093-0.129	0.103	0.096-0.173	0.123
Methionine	0.014-0.023	0.018	0.018-0.027	0.021
Phenylalanine	0.031-0.043	0.037	0.035-0.055	0.043
Proline	0.051-0.08	0.066	0.047-0.075	0.060
Propionate	0.006-0.018	0.009	0.010-0.018	0.014
Pyruvate	0.014-0.075	0.042	0.029-0.062	0.046
Threonine	0.021-0.044	0.037	0.024-0.065	0.042
Trimethylamine N-oxide	0.000-0.000	0.000	0.000-0.055	0.031
Valine	0.176-0.256	0.209	0.178-0.295	0.233

Myo-Inositol	0.012-0.023	0.018	0.016-0.027	0.022
Trans-4-Hydroxy-L-proline	0.015-0.028	0.020	0.017-0.031	0.023

Table S2. Metabolite pairs with a difference of correlation coefficient more than 1 between before and after severe cold stress in Sanhe cattle.

Metabolite1	Metabolite 2	Correlation coefficient (before)	Correlation coefficient (after)	Difference
Creatine phosphate	Methanol	0.78**	-0.82**	1.6
Creatine phosphate	Propionate	0.55	-0.76*	1.31
3-Hydroxyisobutyrate	4-Hydroxyphenylacetate	0.69*	-0.52	1.21
Hippurate	Methionine	0.77**	-0.4	1.17
4-Hydroxyphenylacetate	Methanol	0.65*	-0.52	1.17
Dimethyl sulfone	Propionate	0.82**	-0.29	1.11
4-Hydroxyphenylacetate	Propionate	0.82**	-0.28	1.1
3-Hydroxybutyrate	Betaine	-0.71*	0.39	1.1
3-Hydroxybutyrate	3-Hydroxyisobutyrate	0.75*	-0.33	1.08
Creatine	Creatine phosphate	0.79**	-0.27	1.06
3-Hydroxyisobutyrate	Valine	0.70*	-0.31	1.01
4-Hydroxyphenylacetate	Dimethyl sulfone	0.74*	-0.27	1.01

Notes: *: P-value < 0.05; **: P-value < 0.01.

Table S3. Relevant metabolic pathway by enrichment analysis with 19 differential metabolites in Sanhe cattle.

Metabolic pathways	Total	Expected	Hits	Raw p
Glycine and Serine Metabolism	59	1.09	4	2.01E-02
Betaine Metabolism	21	0.39	2	4.55E-02
Valine, Leucine and Isoleucine Degradation	60	1.11	3	9.49E-02
Fatty Acid Biosynthesis	35	0.65	2	1.35E-01
Propanoate Metabolism	42	0.78	2	1.81E-01
Methionine Metabolism	43	0.80	2	1.88E-01
Glucose-Alanine Cycle	13	0.24	1	2.17E-01
Vitamin K Metabolism	14	0.26	1	2.32E-01
Arginine and Proline Metabolism	53	0.98	2	2.58E-01
Alanine Metabolism	17	0.32	1	2.75E-01
Phosphatidylinositol Phosphate Metabolism	17	0.32	1	2.75E-01
Spermidine and Spermine Biosynthesis	18	0.33	1	2.88E-01

Ethanol Degradation	19	0.353	1	3.02E-01
Glutathione Metabolism	21	0.39	1	3.28E-01
Inositol Phosphate Metabolism	26	0.48	1	3.89E-01
Phenylalanine and Tyrosine Metabolism	28	0.52	1	4.12E-01
Selenoamino Acid Metabolism	28	0.52	1	4.12E-01
Urea Cycle	29	0.54	1	4.23E-01
Inositol Metabolism	33	0.61	1	4.66E-01
Amino Sugar Metabolism	33	0.61	1	4.66E-01
Aspartate Metabolism	35	0.649	1	4.87E-01
Galactose Metabolism	38	0.705	1	5.16E-01
Pyruvate Metabolism	48	0.891	1	6.02E-01
Glutamate Metabolism	49	0.909	1	6.09E-01
Tryptophan Metabolism	60	1.11	1	6.86E-01
Tyrosine Metabolism	72	1.34	1	7.53E-01

Table S4. Networks between metabolites and genes associated with severe cold stress.

Pathway	Num_g	Genes	Num_m	Metabolites	FDR
Metabolism of amino acids and derivatives	8	*RPLP2;HNMT;DLD;RPS18;RPL22L1;CRYM;RARS;MRPL30*	9	3-Hydroxyisobutyrate; Creatine phosphate;Creatinine;Acetate; Valine;Creatine;Betaine;Methanol;Leucine	0.0000501***
SLC-mediated transmembrane transport	1	*SLC30A6*	8	Propionate;Creatinine;Acetate; Valine;3-Hydroxybutyrate;Betaine;Myo-Inositol;Leucine	0.00023***
Transport of bile salts and organic acids, metal ions and amine compounds	1	*SLC30A6*	6	Creatinine; Valine;3-Hydroxybutyrate;Betaine;Myo-Inositol;Leucine	0.000481***
Transport of small molecules	6	*NPC1;FXYD3;EIF2S3;LIPA;LPL;SLC30A6*	8	Propionate;Creatinine;Acetate; Valine;3-Hydroxybutyrate;Betaine;Myo-Inositol;Leucine	0.000833***
Propanoate metabolism	2	*DLD;ECHDC1*	3	Acetate;Methanol;Propionate	0.0318*
Metabolism	19	*NUP98;CHKB;COQ10A;CRYM;GDPD1;HNMT;*	13	3-Hydroxyisobutyrate;Propionate;Creatine phosphate;Creatinine;Acetate;	0.0387*

		DGAT2;DLD;RPS18;RARS;HPGD;RPE;SLC25A20;PFKFB3;LPL;RPL22L1;UQCRFS1;RPLP2;MRPL30		Valine;Trimethylamine N-oxide;Creatine;Betaine;Methanol;Hippurate;Myo-Inositol;Leucine	
Glycine, serine, alanine, and threonine metabolism	2	*NUP98;DLD*	4	Acetate;Creatine;Creatine phosphate;Betaine	0.0387*
Trans-sulfuration pathway	1	*DLD*	3	Creatine;Creatine phosphate;Creatinine	0.0408*
Histidine, lysine, phenylalanine, tyrosine, proline, and tryptophan catabolism	3	*HNMT;DLD;CRYM*	3	Methanol;Valine;Leucine	0.0408*
Amino Acid metabolism	4	*HNMT;DLD;WARS;RARS*	3	Valine;Creatine phosphate;Leucine	0.059
Branched-chain amino acid catabolism	1	*DLD*	3	3-Hydroxyisobutyrate;Valine;Leucine	0.0735
Translation	9	*RPLP2;SRP72;RPS18;RARS;EIF2S3;RPL22L1;EIF2B1;WARS;MRPL30*	2	Valine;Leucine	0.0754
tRNA charging	2	*WARS;RARS*	2	Valine;Leucine	0.0843

Notes: Num_g: number of gene; Num_m: number of metabolites; *: *P*-value < 0.05; **: *P*-value < 0.01.

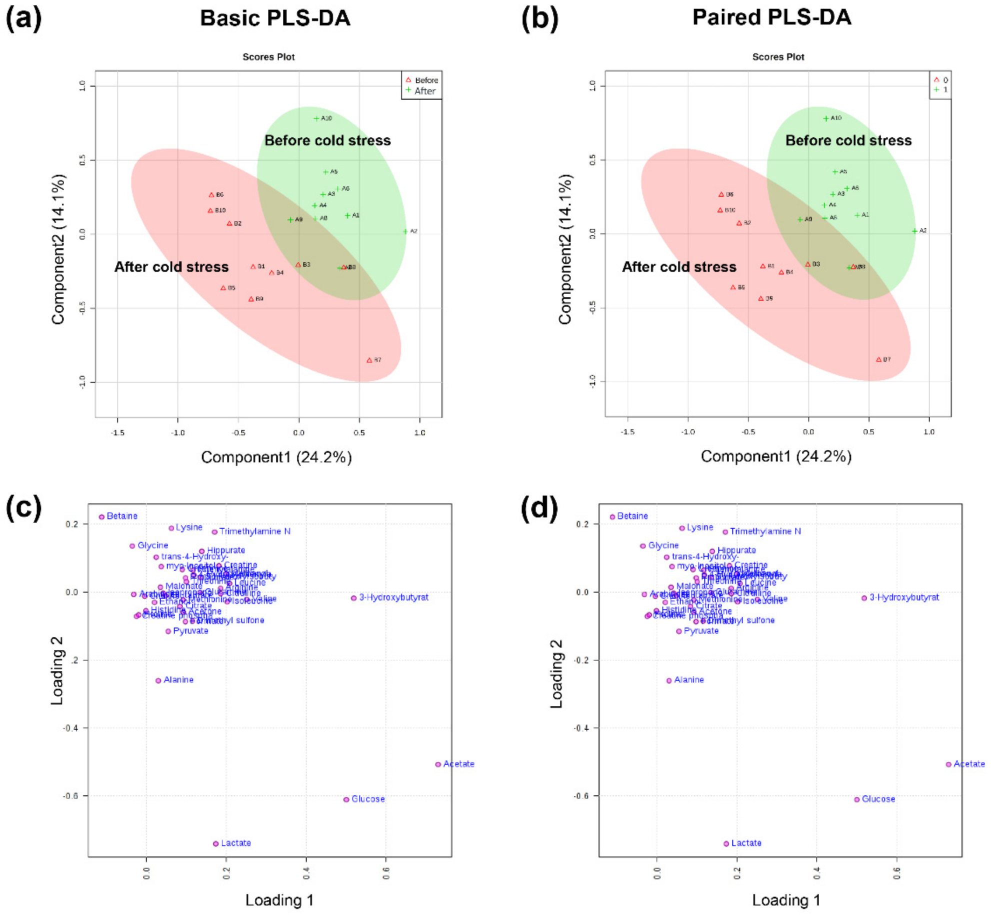

Figure S1. The comparison between standard PLS-DA and paired PLS-DA. (a) The scores plot of standard PLS-DA. (b) The scores plot of paired PLS-DA. (c) The loading plot of standard PLS-DA. (d) The loading plot of paired PLS-DA.
